# X-ray structure and activities of an essential *Mononegavirales L*-protein domain

**DOI:** 10.1038/ncomms9749

**Published:** 2015-11-09

**Authors:** Guido C. Paesen, Axelle Collet, Corinne Sallamand, Françoise Debart, Jean-Jacques Vasseur, Bruno Canard, Etienne Decroly, Jonathan M. Grimes

**Affiliations:** 1 Division of Structural Biology, Wellcome Trust Centre for Human Genetics, Oxford, OX3 7BN UK; 2 AFMB, CNRS, Aix-Marseille University, UMR 7257, Case 925, 163 Avenue de Luminy, Marseille, 13288 France; 3 Department of Nucleic Acids, IBMM, UMR 5247, CNRS, Université Montpellier, ENSCM, Campus Triolet, Place E. Bataillon, Montpellier, 34095 France; 4 Diamond Light Source Limited, Harwell Science and Innovation Campus, Didcot, OX11 0DE UK

**Keywords:** Virology, Structural biology, Viral proteins

## Abstract

The *L* protein of mononegaviruses harbours all catalytic activities for genome replication and transcription. It contains six conserved domains (CR-I to -VI; Fig. 1a). CR-III has been linked to polymerase and polyadenylation activity, CR-V to mRNA capping and CR-VI to cap methylation. However, how these activities are choreographed is poorly understood. Here we present the 2.2-Å X-ray structure and activities of CR-VI+, a portion of human Metapneumovirus *L* consisting of CR-VI and the poorly conserved region at its C terminus, the +domain. The CR-VI domain has a methyltransferase fold, which besides the typical *S*-adenosylmethionine-binding site (^SAM^P) also contains a novel pocket (^NS^P) that can accommodate a nucleoside. CR-VI lacks an obvious cap-binding site, and the ^SAM^P-adjoining site holding the nucleotides undergoing methylation (^SUB^P) is unusually narrow because of the overhanging +domain. CR-VI+ sequentially methylates caps at their 2′*O* and *N*7 positions, and also displays nucleotide triphosphatase activity.

**Supplementary information:**

The online version of this article (doi:10.1038/ncomms9749) contains supplementary material, which is available to authorized users.

## Abstract (web summary)

The*Mononegavirales*include Ebola virus, Rabies, Measles virus and human Metapneumovirus (hMPV). Here, the authors have reported the high resolution crystal structure of a domain of the large protein of hMPV, providing insight into the mRNA modifying activities of this protein.

**Supplementary information:**

The online version of this article (doi:10.1038/ncomms9749) contains supplementary material, which is available to authorized users.

## Graphical abstract

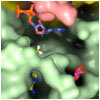

## Introduction

**Figure 1:**
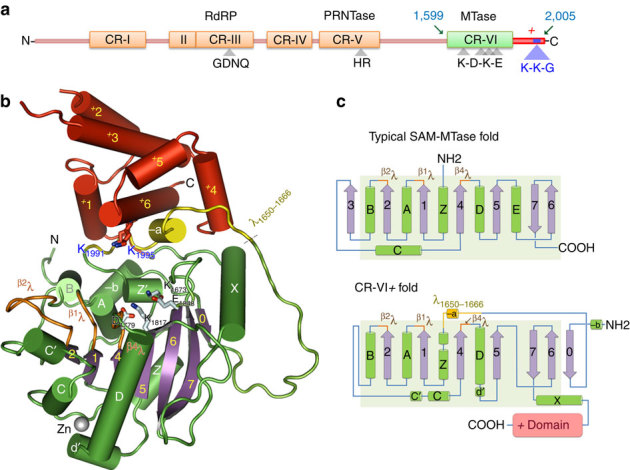
The structure of the MTase domain of hMPV *L.* (**a**) Domain organization of hMPV *L*, with at its C terminus the 46.5 kDa CR-VI+ domain (residues 1,599–2,005), comprising CR-VI (green), which contains the K-D-K-E motif typical for 2′*O*-MTases, and the +domain (red), carrying the K-K-G motif (blue). Boundaries of CR-I to -V are approximate. CR-III contains the G-D-N-Q signature motif for polymerase (RdRP) activity, and CR-V contains the HR motif for PRNTase activity. (**b**) Cartoon representation of the CR-VI+ crystal structure, from amino (N) to carboxy (C) terminus (no structure could be assigned to the first ∼18 residues). The +domain is shown in red, with K_1991_ and K_1995_ of the K-K-G motif in stick format. The CR-VI (MTase) domain is coloured purple (β-strands) and green (helices and loops), except for ^β1^λ, ^β2^λ and ^β4^λ (the loop regions C terminal of β-strands 1, 2 and 4 that form ^SAM^P; orange), and λ_1650–1666_ (which disengages itself from the main CR-VI-fold to interact with the +domain; yellow). Nomenclature of helices and strands follows that used for other MTases (**c**). The pale-blue sticks show the K-D-K-E motif. A Zn-ion (silver sphere) is co-ordinated by H_1766_, H_1798_, C_1802_ and C_1805_. (**c**) Schematic representation of the secondary structure of a prototypical SAM-dependent MTase (top) and of the hMPV CR-VI domain (bottom). Helices are in green, strands in light purple and coils in blue, except for ^β1^λ, ^β2^λ and ^β4^λ (orange) and λ_1650–1666_ (yellow). CR-VI displays some deviations from the prototypical SAM-MTase fold, some of which it shares with other RNA-MTases, including the long N-terminal coil, a longer αD and an extra helix (αX) at the C terminus. αE is absent, whereas, atypical for viral MTases, αB is fully formed. CR-VI, moreover, has an unusual β-sheet; it lacks β3, but this is compensated for by the addition, at the other end of the sheet, of a new strand (β0), which glues the start of the N-terminal coil to the main structure. Also unusual is the fragmentation of αZ (resulting in the small z′-helix).

The *Mononegavirales* order groups five families of monopartite, negative-strand RNA viruses many of which are highly pathogenic and/or contagious; the *Filoviridae* (of which Ebola virus is a representative), the *Bornaviridae* (Borna disease virus), the *Nyamiviridae* (midway virus), the *Rhabdoviridae* (rabies, vesicular stomatitis virus (VSV)) and the *Paramyxoviridae* (measles virus, human metapneumovirus (hMPV)). These viruses encode a large RNA polymerase (*L*) (usually >2,000 amino acids) that is crucial to viral replication (Fig. 1a). It has two distinct roles to replicate the RNA genome and to transcribe viral mRNA. As such it not only polymerizes RNA but also synthesizes fully methylated cap structures^1^. Capping involves the co-transcriptional addition of a guanosine (**G**) to the first nucleotide (N_1_) of the nascent RNA chain via a 5′-5′ triphosphate bridge, resulting in a **G**pppN_1_- structure. Typically, this is followed by methylation of nitrogen 7 (*N*7) of **G**, giving rise to ^m^**G**pppN_1_-, and of the 2′-oxygen (2′*O*) of the N_1_ ribose (^m^**G**pppN_1m_-). The cap protects mRNAs against 5′-exonucleases and promotes RNA transport and translation, while 2′*O*-methylation prevents detection by cellular-immunity sensors^2,3,4^. In *Rhabdoviridae*, CR-V catalyses cap addition by means of an unconventional polyribonucleotydyl-transferase (PRNTase) reaction where a conserved histidine in CR-V forms a covalent phosphoamide bond with the transcript, resulting in a CR-V-pRNA intermediate. The capped transcript is released after ligation of a **G**pp to the pRNA. This mechanism differs from capping in eukaryotes and most other viruses, in which a guanylyltransferase (GTase) forms a phosphoamide bond with **G**p, before transferring it to 5′ppRNA^2^. *Paramyxoviridae* also contain a PRNTase signature motif in their CR-V domains, suggesting they use the same capping strategy as *Rhabdoviridae*. In addition, however, paramyxovirus and filovirus *L* proteins contain a KxxxKxxG sequence (K-K-G motif) at their C termini, reminiscent of a signature motif for eukaryotic GTases, where one of the lysines forms the transient phosphoamide bond with **G**p in the capping reaction^5^. A C-terminal domain of Rinderpest virus *L* (containing CR-VI and the downstream K-K-G motif) was shown to form such a bond^6^, leaving open the possibility that *Paramyxoviridae* use GTase activity for capping.

Cap methylation is catalysed by *S*-adenosylmethionine (SAM)-dependent methyltransferases (MTases), which position a SAM molecule next to the target atom on the RNA, enabling the direct transfer of a methyl group and converting SAM into *S*-adenosylhomocysteine (SAH). In 2′*O*-MTases, a conserved K-D-K-E tetrad potentiates methyl transfer^2^. As yet, the boundaries of CR-VI, the putative MTase of *L*, are not well defined, nor is it clear if CR-VI acts on its own or in conjunction with other domains, whether it mediates both *N*7- and 2′*O*-methylation and which of these methylations would take place first. Research into the activities of *L* and the mechanisms underpinning them has historically been hampered by a complete, order-wide absence of high-resolution structures. To redress this, we set out to study *L* domains of hMPV, a paramyxovirus of the *Pneumovirinae* subfamily closely related to respiratory syncytial virus (RSV). Like RSV, hMPV is highly contagious and causes respiratory tract disease^7^. As a part of this study, we expressed CR-VI+, a 406-residue fragment comprising CR-VI and the adjoining ‘+ domain’, the variable region carrying the K-K-G-motif, investigated its MTase activity and solved its crystal structure. Besides sequentially methylating the 2′*O* and *N*7 atoms of small capped RNAs, CR-VI+ also 2′*O* methylates uncapped substrates and displays nucleotide triphosphatase (NTPase) activity. Both the CR-VI domain, which assumes a fairly standard MTase fold, and the K-K-G motif of the (mainly helical) +domain are required for the MTase reactions. Combined, the data provide new insights into the modification of the 5′-ends of transcripts emerging from the polymerase domain of *L*. This structural information on a mononegavirus *L* protein, and the new insights in the capping mechanism it provides, should spur the development of novel antiviral drugs against this important group of highly pathogenic viruses.

## Results

### MTase activities

**Figure 2:**
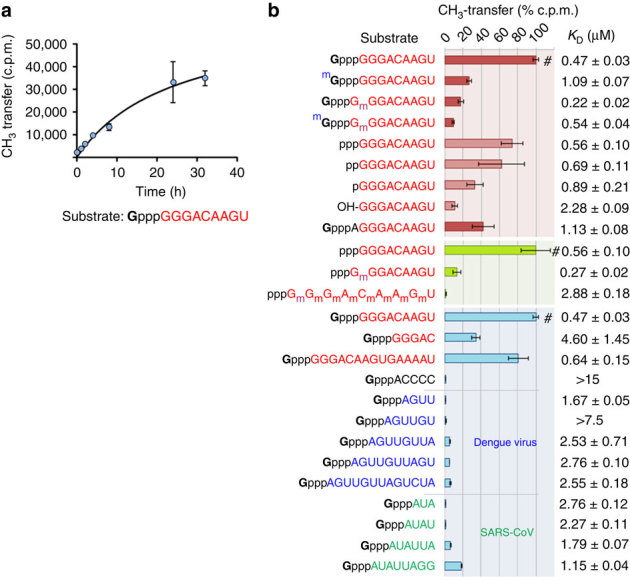
MTase activity of CR-VI+. (**a**). The transfer of tritiated methyl groups from SAM molecules to a capped RNA substrate (**G**pppGGGACAAGU), containing the consensus start sequence of hMPV transcripts (in red), was monitored over time. The rather slow *in vitro* methylation suggests the reaction is impaired compared with the *in vivo* activity of intact *L*, regions of which may aid the methyl transfer (for example, by correctly positioning substrate RNAs to the MTase; see main text). The bars and error bars correspond to the mean values from three independent measurements and their s.d.’s, respectively. (**b**). Substrate specificity was determined as above, but using various synthetic RNA substrates, and allowing the reactions to proceed for 16 h. Substrates were compared with **G**pppGGGACAAGU (red and blue panels) and pppGGGACAAGU (green panel), for which the degree of methylation was set at 100% (#, marked bars). The red-shaded panel compares the degree of methylation of nine-nucleotide-long hMPV start sequences with different 5′-ends and methylation states (the lighter bars represent uncapped RNAs). The results indicate efficient methylation of RNAs that already carried a (cold) methyl group, either at their *N*7-guanine or 2′*O*-ribose position (confirming the occurrence of 2′*O*- and *N7*-methylation, respectively), and of uncapped RNAs (especially pppRNA). The pppRNA substrate is predominantly methylated at N_1_, as substrates that were methylated beforehand at this nucleotide do not undergo substantial additional methylation (green panel). CR-VI+ prefers the hMPV start sequence over the short **G**pppACCCC sequence, and over RNA-start sequences of Dengue virus and SARS coronavirus, irrespective of their lengths (blue panel). A nine-nucleotide hMPV substrate, however, is much preferred over one with only five nucleotides, indicating that additional interactions take place between the protein and nucleotides 6–9. Consistently a 10-times lower *K*_D_ (dissociation constant) characterizes the interaction of CR-VI+ with the 9-mer, compared with that with the 5-mer. The *K*_D_s, measured in triplicate using a dot-blot assay and listed at the right of the diagram (±s.d.’s), also show that capped and uncapped hMPV sequences are bound with comparable affinities, and that 2′*O*-methylated substrates are preferred over unmethylated ones.

**Figure 3:**
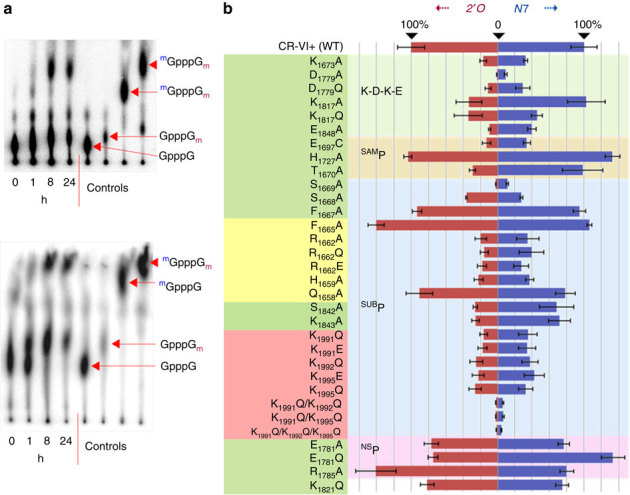
*N*7- and 2′*O*-methylation. (**a**) Thin-layer chromatograms. Following CR-VI+-mediated methyl transfer from SAM onto **G**pppGGGACAAGU (in which **G** was [^32^P]-labelled), nucleotides 2–9 were removed by nuclease P1 digestion, and the caps were separated by thin-layer chromatography (TLC). The controls (**G**pppG, **G**pppG_m_, ^m^**G**pppG and ^m^**G**pppG_m_, from left to right) were obtained with the same substrate, using MTases that specifically methylate caps at the *N*7 or 2′*O* positions (human *N*7- and vaccinia virus 2′*O*-MTase). The TLC experiment used 0.65 M LiCl as mobile phase, allowing a clear separation of ^m^**G**pppG and ^m^**G**pppG_m_ (top). The caps on the TLC plate were subsequently further resolved, this time using 0.45 M (NH_2_)_2_SO_4_ as mobile phase for a better separation of **G**pppG and **G**pppG_m_ (bottom). **G**pppG_m_ appears first (after a 1-h incubation), ^m^**G**pppG_m_ becomes prominent at a later stage, and ^m^**G**pppG was not observed, indicating that 2′*O*-methylation of N_1_ precedes *N*7-methylation of **G**. (**b**). The effect of point mutations on the MTase activities of CR-VI+, measured after a 16-h incubation period (by means of a filter-binding assay, as in Fig. 2), using **G**pppGGGACAAGU substrates that were methylated beforehand at *N*7 of **G** or 2′*O* of N1, to specifically monitor 2′*O* or *N*7-MTase activities, respectively. Mutants are listed against a yellow, green or red background, to indicate that the altered residue belongs to λ_1650–1666_, the rest of the CR-VI domain, or the +domain, respectively. They are also grouped according to whether they change the K-D-K-E tetrad, ^SAM^P, ^SUB^P or ^NS^P. The results highlight the importance of essential ^SUB^P residues (such as the K-K-G lysines K_1991_, K_1992_ and K_1995_ and λ_1650–1666_ residues H_1659_ and R_1662_) for both 2′*O*- and *N*7-methylation. All tetrad residues are crucial for 2′*O*-methylation, while D_1779_ in particular is important for *N7*-MTase activity. The bars and error bars correspond to the mean values from three independent measurements and their s.d.’s, respectively.

*In vitro*, CR-VI+ most effectively binds and methylates synthetic RNAs containing the conserved start sequence of hMPV transcripts, preferring a substrate length of nine nucleotides (Fig. 2). The methylation occurs at the **G** *N*7 and N_1_-2′*O* positions (Figs 2 and 3a), with 2′*O*-methylation preceding *N*7-methylation (Fig. 3a), an uncommon order of events also occurring in VSV^8^. CR-VI+, moreover, efficiently methylates uncapped RNAs with 5′-phosphate groups (especially pppRNA), primarily at the 2′*O* atom of N1 since almost no ^3^H-methyl transfer takes place onto the pppG_m_GGACAAGU substrate where this atom is blocked (Fig. 2b, green bars). The 2′*O*-methylation of pppGGGACAAGU represents ∼50% of that observed with **G**pppGGGACAAGU (which has an extra methylation target in the **G** *N*7 atom; Fig. 2b, red bars). Mutagenesis studies show that all K-D-K-E residues (K_1673_, D_1779_, K_1817_ and E_1848_) are essential for 2′*O*-methylation, while the aspartic acid (D_1779_) in particular is important for *N*7-methylation (Fig. 3b), as was also observed in *Flaviviridae* MTases^9^. Other residues, many of which belong to the +domain rather than the core MTase fold, were also found to be essential for the MTase activities (the ‘^SUB^P’ residues in Fig. 3b, described below).

### Structure of the +domain

The crystal structure of CR-VI+ was solved to 2.2 Å resolution in space group P2_1_2_1_2_1_ with two molecules, disulphide-linked at residue C_1877_, in the asymmetric unit, assuming a ‘head-to-toe’ conformation (Supplementary Fig. 1). There are no significant differences between the molecules, which have a slightly twisted, bi-lobed shape, composed of two globular domains, the larger corresponding to CR-VI (residues ∼1,616–1,883) and the smaller to the +domain (1,884–2,005; Fig. 1b). Although composed of only ∼120 residues in *Pneumovirinae*, the size of the +domain varies greatly within the *Mononegavirales* order, reaching ∼240 residues in *Rhabdoviridae*. In hMPV, it consists of six α-helices (α^+^1–6). Helix α^+^6 contains the K-K-G motif (Supplementary Fig. 2) and together with α^+^1 leans over the active cleft of CR-VI. Helix α^+^4, and to a lesser extent helices α^+^1, α^+^5 and α^+^6, packs down on λ_1650–1666_, the second half of a long loop (residues 1,635–1,666) that swerves around the CR-VI domain (Fig. 1b and Supplementary Fig. 3; ‘λ’ is used throughout the paper to denote loops). The λ_1650–1666_ region, which contains a small helix (α-a), acts as a fulcrum allowing the +domain to pivot relative to the CR-VI domain. Helix α^+^3 varies in length, from 4½ turns (in most crystals) to 6 (in Protein Data Bank (PDB) 4UCY), and the loop between α^+^2 and α^+^3 is always disordered (Supplementary Fig. 3). In crystals of the monomeric C_1877_A mutant (space group P3_1_21), this disordered, unstable region is further enlarged, as helix α^+^2 completely unfolds and α^+^3 unwinds to 3½ turns.

### Structure of the CR-VI domain

**Figure 4:**
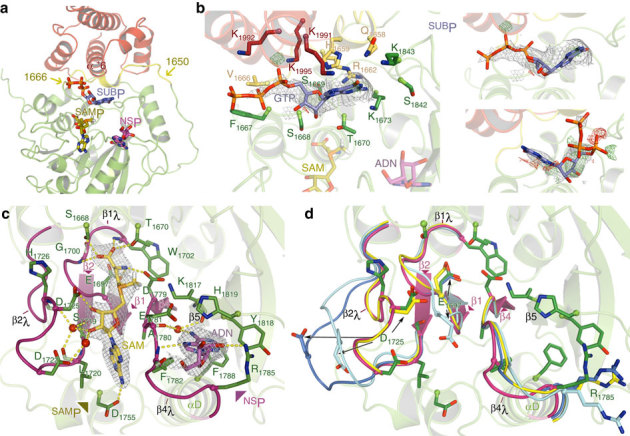
CR-VI+-binding pockets. The cartoon representations show the +domain in red and CR-VI in green, with λ_1650–1666_ in yellow. SAM (in ^SAM^P), GTP (in ^SUB^P) and adenosine (ADN; in ^NS^P) are shown as sticks, with the C atoms coloured gold, slate and magenta, respectively. Hydrogens (in white) accentuate the methyl group of SAM. 2Fo-Fc electron density maps around the ligands are represented in grey mesh (contoured at 1σ). (**a**) The relative positions of the pockets in the protein. (**b**) Close-up of ^SUB^P, which is defined by residues of the +domain (particularly the K-K-G motif), λ_1650–1666_ and the CR-VI domain. Residues involved in ligand binding are shown as sticks. GTP is fitted in different orientations into the density in the PDB 4UCZ structure (main figure, and top figure to the right, where the guanosine ring is turned 180°) and in the PDB 4UCI structure (bottom right, where the ligand lays in the opposite direction), highlighting that the ligand can bind in different orientations within the spacious pocket. (**c**) ^SAM^P and ^NS^P containing a SAM and ADN ligand, respectively (PDB 4UCI, in which ^SUB^P is also occupied). Residues lining the pockets are shown as sticks. The loops delineating ^SAM^P (^β1^λ, ^β2^λ and ^β4^λ) and the β-strands they originate from are shown in magenta. The dashed yellow lines show putative hydrogen bonds. (**d**). Superposition of three other CR-VI+ structures onto that in **c**, highlighting the flexibility of ^β1^λ (especially E_1697_), ^β2^λ and ^β4^λ. The structure in blue (PDB 4UCK) contains SAM, whereas those in yellow (4UCL) and aquamarine (4UCJ) have empty ^SAM^Ps (this suggests that there is no strict correlation between ^SAM^P occupancy and the position of ^β2^λ). ^NS^P is empty in the three superposed structures, which apparently affects the position of their ^β4^λ loops and especially of the R_1785_ side group, which closes the pocket when occupied. All overlaid structures have empty ^SUB^Ps.

The CR-VI domain shares some peripheral characteristics with the 2′*O*-MTases of SARS coronavirus (PDB: 2XYQ)^10^, vaccinia virus (1VP3) (ref. 11) and bluetongue virus (VP4-subunit; 2JHA)^12^, such as the long N-terminal loop and the position of helix αX. In its active core, however, it better resembles RrmJ-type flavivirus MTases (for example, 3EVF^9^; Supplementary Fig. 4). Most notably, hMPV and flaviviruses share an unusually long (∼10 residues), flexible ^β2^λ (that is, the loop immediately following the β2-strand), which forms the SAM-binding pocket (^SAM^P) along with loops ^β1^λ and ^β4^λ, shielding it from the solvent (Fig. 1b, c). In Wesselsbron (flavi-)virus, ^β2^λ is found in closed or open conformations, either packing up against SAM (PDB: 3ELW) or exposing it to the solvent (3EMB)^13^, changes that may assist SAM uptake and/or SAH expulsion. In CR-VI+, ^β2^λ similarly assumes alternate conformations (Fig. 4c,d). In the ‘closed’ form, the ligand’s ribose group is hydrogen bonded to D_1725_, and—via a water—to D_1722._ Loops ^β1^λ and ^β4^λ also show a degree of flexibility. ^β1^λ residue E_1697_, conserved in paramyxo- and filoviruses, forms a hydrogen bond with the NH_2_ group of SAM and is essential for both MTase activities (Fig. 3b). However, in the absence of SAM, its side chain either moves into the sub-pocket that normally accommodates the NH_2_ group, or, more markedly, turns towards the solvent in the direction of ^β2^λ, which in this case assumes the ‘open’ position (Fig. 4d). ^β4^λ forms a side wall of ^SAM^P, and together with αD and β5 also defines a deep, hydrophobic cavity not present in other MTases, termed ^NS^P (or nucleoside-binding pocket). Although a role for ^NS^P has yet to be determined, the pocket binds the adenosine moiety of SAM or ATP, soaked at high (25 mM) concentrations into CR-VI+ crystals, causing ^β4^λ to impinge onto ^SAM^P (Fig. 4c,d; when SAM is used for soaking, one SAM molecule occupies ^SAM^P, and another binds to ^NS^P). GTP was not observed in ^NS^P, but this may reflect a lower soaking concentration (2.5 mM), due to GTP’s poor solubility. With the exception of E_1781_, the amino acids lining ^NS^P are poorly conserved beyond the *Pneumovirinae*, and mutating key ^NS^P residues barely affects the MTase activities (Fig. 3b). CR-VI, finally, contains a Zn-finger, which is not conserved beyond the *Pneumovirinae* subfamily and links the small α–d′ to the rest of the structure (Fig. 1b).

**Figure 5:**
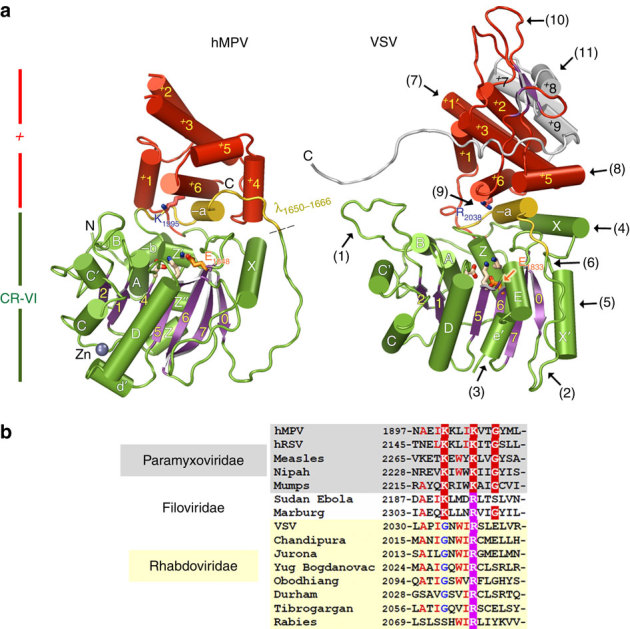
Comparison of the CR-VI+ domains of hMPV and VSV. (**a**). Cartoon representations. The CR-VI domains are similar, and share the unusual, strand-0 containing β-sheet (purple), the rather large ^β2^λ—indicated by arrow (**1**)—and the long N-terminal loop (**2**), which runs somewhat differently in VSV. The λ_1650–1666_ peptide on which the +domain rests (yellow) also has a homologue in VSV. The Zn-finger, however, is not conserved, and α-helices B and Z are not fragmented. Helix αE, an element of the standard MTase topology (Fig. 1c), is present in VSV (**3**), as a result of which ^NS^P may have disappeared. αX is at a different location (**4**), and is preceded by an extra helix (αx′(**5**)). E_1833_, expected to belong to the K-D-K-E tetrad from sequence alignments, is buried in the structure and does not reach the surface of the catalytic pocket (**6**), and the position normally taken by the K-D-K-E glutamate is occupied by T_1831_. The +domain of VSV is tilted, compared with that of hMPV, and more elaborate. Helices α^+^1, α^+^2, α^+^3, α^+^5 and α^+^6 are conserved, but the α^+^1–α^+^2 loop is replaced by an extra helix (α^+^1′ (**7**)). α^+^4 is absent, whereas α^+^5 is enlarged and immediately follows α^+^3 (**8**). The 2-residue loop connecting α^+^5 and α^+^6 in hMPV is replaced by a 34-residue coil carrying a small three-stranded β-sheet (**9**). Helix α^+^6 seems best conserved between the two +domains, although a K-K-G motif is not present in VSV. However, R_2038_, which is strictly conserved in *Rhabdoviridae*, takes the place of K_1995_ ((**10**) and alignment below). In VSV, the +domain is extended beyond α^+^6 with a 65-residue, partly helical, but mainly unstructured polypeptide (in grey (**11**)). Colour scheme and labelling are as in Fig. 1b. (**b**). Alignment of α^+^6-helices from Mononegavirales *L* proteins. K-K-G motif residues are highlighted in red; the arginine replacing the second lysine of the motif in *Filoviridae* and *Rhabdoviridae* is highlighted in magenta. Red letters indicate other (less strictly) conserved residues, except for the G that replaces the first lysine of the K-K-G motif in most *Rhabdoviridae* (blue).

During the preparation of this manuscript, a 3.8-Å structure of VSV *L*, obtained by electron cryo-microscopy, was published^14^. The CR-VI (or MTase) part of VSV resembles that of hMPV, but apparently lacks a deep ^NS^P pocket (PDB: 5a22, Fig. 5). The +domain (C-terminal domain) is more elaborate in VSV than in hMPV, containing extra regions N and C terminal of helix α^+^6. The helix itself, however, appears well conserved, both in length and in position. Although a K-K-G motif is not present in VSV, it does contain an arginine (R_2038_) strictly conserved among the *Rhabdoviridae*, which is structurally equivalent to hMPV’s K_1995_, the second lysine of K-K-G motif.

### The absence of a classical cap-binding site

**Figure 6:**
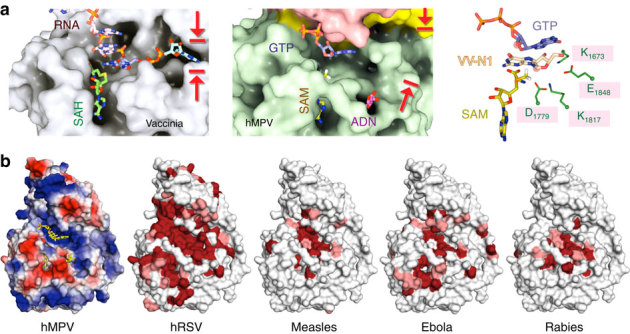
RNA-binding site comparisons. (**a**). Comparison of the RNA-binding sites in vaccinia virus cap-MTase (PDB 1AV6) and CR-VI+. The vaccinia virus MTase (white surface, left) has a narrow cap-binding pocket (in between the red arrows) and a large, open RNA-binding site (adjoining the SAH-containing ^SAM^P). In CR-VI+ (coloured surface, middle) the cap-binding pocket is not present, whereas the RNA-binding site is narrowed (into ^SUB^P) by the +domain overhang (in red). A structural superposition (obtained by aligning the K-D-K-E tetrads, right) shows that the GTP ligand in CR-VI+ is situated at a considerably greater distance from the tetrad than the first transcribed nucleotide (N_1_) in the vaccinia virus MTase–RNA complex (shown in light pink). The 2′*O* atoms of the nucleotides are shown as transparent, red spheres. (**b**). ^SUB^P conservation within the Mononegavirales order. The surface presentation on the left shows the basic (blue) and acidic (red) charge distribution on CR-VI+. The ligands are in yellow. The other cartoons show the surface of the hMPV CR-VI+ domain in the same orientation, but in white. Residues that are conserved in the hRSV, Measles, Ebola or Rabies virus homologues of CR-VI+ are coloured dark red (identical residues) or pink (similar residues), and cluster around ^SUB^P and ^SAM^P.

A common feature of MTases involved in cap methylation is a defined cap-binding pocket that binds **G** with high affinity, enabling subsequent, low-affinity interactions with the triphosphate bridge and the first few nucleotides, thus precluding methylation of uncapped RNAs^15,16^. In CR-VI+, however, an open, solvent-exposed area is found where this pocket is normally located (Fig. 6a). Moreover, **G**pppG- or ^m^**G**pppG binding was not observed in co-crystallization or soaking experiments, suggesting that CR-VI+ has a weak affinity for **G** at best and that the cap is not required for substrate recognition. This is consistent with CR-VI+ binding capped and uncapped RNAs with similar strength and being able to 2′*O*-methylate uncapped RNAs (Fig. 2b). Strong binding would also prevent translocation of **G** into ^SUB^P for *N*7-methylation. Although a high-affinity cap-binding site is clearly absent from CR-VI+, the low-affinity nucleoside binding to ^NS^P and the convenient location of this pocket relative to ^SUB^P suggest it could provide space for **G** without forming strong interactions (Supplementary Fig. 5).

The absence of a high-affinity cap-binding pocket appears partly compensated for by the narrowing of the groove that in related MTases accommodates the first few nucleotides of the transcripts, by the overhanging +domain (Figs 4 and 6a). In particular, the site adjoining ^SAM^P, which holds the nucleotides undergoing methylation, has become a more elaborate, but narrower and possibly therefore, higher-affinity substrate-binding pocket (termed ^SUB^P as it accommodates the nucleotide undergoing methylation). Consistently, electron density is found in ^SUB^P following soaking or co-crystallization with GTP, whereas in other MTases added GTP predominantly shows up in the cap-binding pocket. In particular, helix α^+^6 and the +domain-affiliated λ_1650–1666_ help shape ^SUB^P through the side chains of K_1991_ and K_1995_ (of the K-K-G motif), and of H_1659_ and R_1662_, respectively (Fig. 4b). The marked decrease in MTase activity of mutants altered at these residues (Fig. 3b) illustrates the importance of ^SUB^P in correctly presenting the substrate nucleotides to SAM. Nevertheless, the pocket is too spacious for a single nucleotide, and the electron density in ^SUB^P from a number of soaked crystals suggests that bound GTP often assumes more than one orientation. In structures where GTP could be fitted with confidence, the guanosine moiety predominantly interacts with λ_1650–1666_ residues H_1659_ and R_1662_, and with K_1991_ and (K-D-K-E residue) K_1673_, which clamp the guanine (Fig. 4b). Unusually for cap-MTases, K_1673_ is not part of αZ, but instead resides on the small z′ (3_10_)-helix (Fig. 1b,c). Whether any of the observed positions of GTP reflects *in vivo* binding of N_1_ (as part of a transcript) is unclear; in MTase–RNA complexes (PDB: 1AV6 (ref. 17), 4N49 (ref. 16)), N_1_ is situated much closer to the K-D-K-E tetrad (Fig. 6a).

### A potential role of CR-VI+ in cap addition

K-K-G residue K_1995_ corresponds to the **G**p binding lysine in the signature motif of eukaryotic GTases, but here is in a loop instead of an α-helix (PDB: 3S24 (ref. 18), 1CKN (ref. 19)) and appears ideally placed to target the phosphate tail of GTP. Although incubation of CR-VI+ with [α-^32^P]- and [β-^32^P]-GTP, in the absence of Mg^++^, resulted in radioactive protein bands on denaturing SDS gels, the level of radioactivity was low, and was not diminished by acid treatment before SDS–polyacrylamide gel electrophoresis (SDS–PAGE), implying it is not due to phosphoamide bond formation. A second, strong argument against a GTase-based capping mechanism in hMPV (and in favour of a PRNTase-based one) is the fact that in the closely related RSV the cap is formed by **G**pp ligation to pRNA^20^.

**Figure 7:**
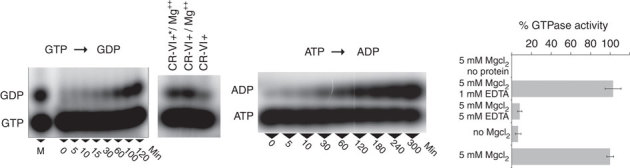
NTPase activity. Autoradiographs of urea–PAGE gels show CR-VI+-mediated conversion of radiolabelled GTP to GDP and ATP to ADP, over time. The smaller autoradiograph shows the requirement of Mg^++^ for the reaction (allowed to proceed for 1 h), and also shows efficient GDP generation by washed, dissolved CR-VI+ crystals (CR-VI+*), dispelling the possibility that the activity is due to contaminants. The diagram on the right (obtained by phosphorimage analysis following electrophoresis) further illustrates the requirement of Mg^++^ and shows the effect of the metal-ion chelator EDTA on the GTPase reaction. The bars and error bars correspond to the mean values from three independent measurements and their s.d.’s, respectively.

We observed that CR-VI+ also displays NTPase activity, converting GTP into GDP and ATP into ADP (Fig. 7). GTPase activity, which is required for PRNTase-based capping, was previously reported in Mononegavirales *L* (ref. 21), but as yet could not be linked to a specific domain within the protein. The reaction observed with CR-VI+, however, is quite slow, possibly because other parts of *L*, or other co-factors, are needed for full activity. In line with this, we were not able to identify key active-site residues. Using the mutants listed in Fig. 3b, the greatest reductions in GTPase activity were obtained with E_1697_C (corresponding to the flexible residue at the bottom of ^SAM^P; Fig. 4d) and S_1669_A (part of ^SUB^P, Fig. 4b) to 54 (±20) and 57 (±16) % of the wild-type activity, respectively (*n*=3). The deletion of the dipeptide G_1645_K_1646_ from the long N-terminal loop resulted in a somewhat more pronounced ∼70% reduction (Supplementary Fig. 6). The NTPase activity was confirmed using crystallized CR-VI+ (Fig. 7).

## Discussion

As a GDP is transferred onto a PRNTase-bound pRNA intermediate during cap synthesis in VSV^21^, the presence of NTPase activity in CR-VI+ would suggest that this domain is involved in cap addition. It is unclear from the structure of VSV *L* how capping, cap methylation and RNA synthesis are coordinated^14^, but an involvement of CR-VI+ in cap addition is consistent with the dynamic nature of the multi-domain polymerases of RNA viruses in general, exemplified by flu where the C-terminal two-thirds of PB2 has been shown to be extremely mobile^22,23^. The presentation of uncapped (but CR-V-linked) pRNA to CR-VI+ would also explain why hMPV *L* does not require a high-affinity cap-binding site. The integrity of both the PRNTase and K-K-G motifs, moreover, has been shown to be essential for viable mRNA synthesis in human parainfluenzavirus 2 (hPIV-2)^5^, in line with capping involving a concerted action of CR-V and CR-VI+. Assuming the transcript is presented to CR-VI+ by the PRNTase, 2′*O*-methylation may actually occur before the cap is added (as a cap is not required for the reaction; Fig. 2b), which would also be consistent with 2′*O*-methylation preceding *N*7-methylation. The possibility that 2′*O*-methylation precedes cap addition was also suggested by the ability of virions of the rhabdovirus spring viremia of carp virus to synthesize uncapped, N1-methylated oligonucleotides^24^. We did not observe significant methylation of free GTP by CR-VI+ (levels were <5% of that of **G**pppGGGACAAGU methylation), suggesting **G** *N*7-methylation takes place after **G** is added to the RNA. In addition 2′*O*-methylated substrates (**G**pppG_m_GGACAAGU and pppG_m_GGACAAGU) show an ∼2-fold higher affinity for CR-VI+, compared with their unmethylated counterparts (Fig. 2b), which presumably reflects an RNA repositioning mechanism allowing the **G** *N*7 atom access to the SAM methyl donor in the second methylation step.

In conclusion, CR-VI+ is a dynamic part of the *L* protein that potentially completes PRNTase-initiated cap addition, and methylates the cap at its 2′*O* and *N*7 positions. The ^SUB^P pocket, which is conserved among *Paramyxoviridae* and *Filoviridae* (Fig. 6b), is clearly instrumental in key activities of the domain, and thus represents an attractive target for the structure-based design of (potentially broad-spectrum) antiviral compounds.

## Methods

### Cloning and expression

The sequence encoding CR-VI+ was PCR amplified using primers that added a C-terminal SGHHHHHH-tag to the translation product, from a synthetic hMPV *L* gene (*hMPV isolate 00-1*, GenBank: AF371337.2), codon optimized for expression in *Spodoptera frugiperda* (*Sf*) cells (Geneart). The amplicon was cloned into pOPIN-E for expression in HEK293T mammalian cells (following transfection using Lipofectamine 2000; Invitrogen), in BL21 Star bacteria (Invitrogen) and in *Sf*21 (insect cells) following co-transfection with (flashBACULTRA) baculovirus (Oxford Expression Systems) using Cellfectin II (Invitrogen)^25^. Mutants were generated by PCR, using primers carrying the mutation (Supplementary Fig. 7). The CR-VI+ DNA was used as a template for mutagenesis, except for the K_1991_Q/K_1995_Q and K_1991_Q/K_1992_Q/K_1995_Q mutants, which were obtained using the K_1995_Q DNA. For each mutation, the forward primer was combined with a vector-specific reverse primer (5′-AGTGGTATTTGTGAGCCAGG-3′), and, in a second PCR, the reverse primer was used together with a vector-specific forward primer (5′-CCTTTAATTCAACCCAACAC-3′). The two PCR products thus generated were digested with either BspQI or BtsI (New England Biolabs), restriction enzymes that cut within the CR-VI+-specific primer regions. The amplicons were then rejoined using T4 DNA ligase (New England Biolabs), and the ligation products were PCR amplified using the vector-specific primers for insertion in the pOPIN-E vector.

### Purification

Seventy-two hours after infection of the suspension cultures with recombinant baculovirus, *Sf*21 cells were spun down (1,000*g*, 7 min, 22 °C) and lysed in one-fourth volume of buffer L (1.5% triton X-100, 5% glycerol, 50 mM arginine, 300 mM KCl, 10 mM imidazole and 20 mM Tris (pH 8.0)). After clarification (10,000*g*, 25 min, 4 °C), the lysate was incubated with benzonase (Novagen; to 0.2 U ml^−1^) and NiNTA resin (Qiagen; 0.3 ml l^−1^ culture) for 5 h at 4 °C, with gentle shaking. The beads were transferred to a 10-ml column and washed with 3 × 10 ml buffer W (20 mM Tris (pH 8.0), 1.5 M NaCl, 10 mM imidazole and 7.5% glycerol) and 1.5 ml of 0.1 M arginine (pH 8.0). Protein was eluted in 0.8 M arginine (pH 8.0). An equal volume of ice-cold 3 M AmSO_4_ was added to the eluent, and the precipitated protein collected (10,000*g*, 10 min, 4 °C). Protein pellets were stored at −20 °C. A similar lysis and purification protocol was used to obtain recombinant protein from HEK293T cells. BL21 Star cells were lysed using Bugbuster Protein Extraction Reagent (Novagen). Expression and purification were monitored by SDS–PAGE and western blotting. Blots were developed with a 1/10,000 dilution of an anti-histidine tag antibody (Penta·His Antibody, Qiagen, catalogue number 34660). Supplementary Fig. 1 shows purified protein following SDS–PAGE under reducing and non-reducing conditions. An uncropped western blot showing the unreduced, insect-cell-expressed protein next to a BenchMark (Invitrogen) molecular weight marker is also shown.

### Selenomethionine incorporation

Twenty hours following infection of an *Sf*21 suspension culture (27.8 °C, with an agitation speed of 130 r.p.m./0.25*g*), cells were collected (70*g*, 10 min, 22 °C) and resuspended in cysteine- and methionine-free SF900II medium (Gibco) supplemented with dialysed fetal bovine serum (Gibco; 7% v/v) and 150 mg l^−1^ L-cysteine (Sigma). Following an additional 4 h at 27.8 °C in the shaking incubator, 250 mg l^−1^ selenomethionine (Sigma) was added. Protein expression was allowed to continue for another 48 h. Proteins were purified as above.

### Crystallization, structure solving, refinement and validation

Crystallization was carried out by vapour diffusion at 20.5 °C using 96-well sitting drop plates (Greiner)^26^. Protein pellets were dissolved in water to 5–6 mg ml^−1^, and initial crystals were obtained by equilibrating 100 nl of protein with 100 nl of reservoir solution C11 of the PGA-HT screen (Molecular Dimensions; pH 6.5) supplemented with guanidine hydrochloride (to 0.1 M), against 0.1 ml of reservoir. Glycerol was added (to 20% v/v) for cryoprotection. Diffraction data were collected at 100 K on Diamond beamlines I02, I03, I04 and I24 (Harwell, UK), and processed using the Xia2 programme suite^27^. A single-wavelength anomalous dispersion experiment allowed determination and refinement of the positions of selenium atoms, as well as calculation of the phases with autoSHARP^28^. An initial model was obtained using MR-ROSETTA^29^, enabling manual model building using COOT^30^. Refinement was performed using autoBUSTER^31^, and validation employed COOT and Molprobity^32^. Molecular replacement (using PHASER^33^) was used to solve additional CR-VI+ structures. Refinement statistics are given in Table 1, and a portion of the electron density map is shown in Supplementary Fig. 8.

**Table 1:**
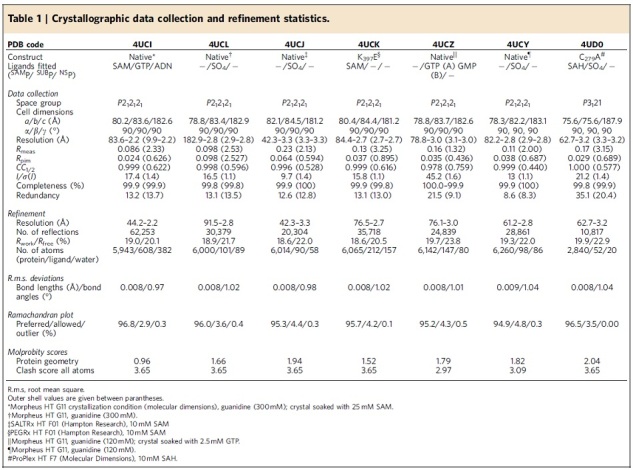
Crystallographic data collection and refinement statistics.

### Synthesis of RNA substrates

RNA sequences were chemically synthesized on a solid support using an ABI 394 synthesizer. After RNA elongation with 2′*O*-pivaloyloxymethyl phosphoramidite monomers^34,35^ (Chemgenes, USA), the 5′-hydroxyl group was phosphorylated and the resulting *H*-phosphonate derivative^36^ oxidized and activated into a phosphoroimidazolidate derivative to react with either phosphoric acid (for ppRNA synthesis), pyrophosphate (pppRNA)^36^ or guanosine diphosphate (**G**pppRNA)^37,38^. *N*7-methylation of the purified **G**pppRNA was performed enzymatically using *N*7-hMTase^37,38^. To prepare monophosphate RNA (pRNA), the 5′-*H*-phosphonate RNA was treated with a mixture of *N*,*O*-bis-trimethylacetamide (0.4 ml), CH_3_CN (0.8 ml) and triethylamine (0.1 ml) at 35 °C for 15 min, and then oxidized with a *tert*-butyl hydroperoxide solution (5–6 M in decane, 0.4 ml; 35 °C, 15 min). After deprotection and release from the solid support, RNA sequences were purified by IEX-HPLC (>95% pure) and their identity were confirmed by MALDI-TOF (Matrix-Assisted Laser Desorption/Ionization Time-of-Fight) spectrometry.

### MTase activity assays

These were performed by combining 4 μM CR-VI+ with 0.7 μM of the purified and validated synthetic RNAs, 10 μM SAM and 0.33 μM ^3^H-SAM (Perkin Elmer) in 40 mM Tris-HCl (pH 8.5) and 1 mM dithiothreitol (DTT). Reactions (at 30 °C) were stopped by a 10-fold dilution in 100 μM ice-cold SAH and the samples were transferred to DEAE filtermats (Perkin Elmer) using a Filtermat Harvester (Packard Instruments). The RNA-retaining mats were washed twice with 10 mM ammonium formate (pH 8.0), twice with water and once with ethanol. They were then soaked with liquid scintillation fluid, allowing the measurement of ^3^H-methyl transfer to the RNA substrates using a Wallac MicroBeta TriLux Liquid Scintillation Counter^13^.

Methylation of GTP was determined in the same buffer (minus the ^3^H-SAM and RNA), using the EPIgeneous methyltransferase assay kit (Cisbio), which measures the generation of SAH, as it competes with d2-coupled SAH for binding to a Lumi4-Tb-labelled anti-SAH antibody, affecting the TR-FRET signal between these compounds. In practice, MTase reactions were stopped after 16 h by addition of the detection reagents, and 1 h later the TR-FRET signal was monitored using a PHERAstar Flashlamp plate reader.

### Thin-layer chromatography analysis of cap structures

G*pppGGGACAAGU (in which the asterisk indicates the [^32^P]-labelled phosphate) was synthesized by incubating pppGGGACAAGU (10 μM) with vaccinia virus capping enzyme (New England Biolabs) in the presence of 1.65 μCi [α-^32^P]-GTP (Perkin Elmer). The capped RNA was purified by precipitation in 3 M sodium acetate supplemented with 1 μg μl^−1^ of glycogen (Thermo Scientific), and submitted to methylation by CR-VI+ (as above), after which it was precipitated again (stopping the reactions), and digested with 1 U of Nuclease P1 (US Biologicals) in 30 mM sodium acetate (pH 5.3), 5 mM ZnCl_2_ and 50 mM NaCl (4 h, 37 °C). The products were spotted onto polyethylenimine cellulose thin-layer chromatography plates (Macherey Nagel), and resolved in two steps, first using 0.65 M LiCl, then 0.45 M (NH_2_)_2_SO_4_ as mobile phase. The radiolabelled caps released by nuclease P1 were visualized using a Fluorescent Image Analyzer FLA3000 (Fuji) phosphor-imager.

### Characterization of RNA-CR-VI+ interactions

Using T4 RNA ligase 1 (20 units; New England Biolabs), [5′-^32^P]-pCp (1.1 μCi) was ligated to the 3′-ends of the RNA substrates (10 μM) in 50 mM Tris-HCl (pH 7.8), 10 mM MgCl_2_, 10 mM DTT and 1 mM ATP (16 °C, overnight). Ligase was removed by RNA precipitation in 3 M sodium acetate supplemented with 1 μg μl^−1^ of glycogen (Thermo Scientific). The radiolabelled RNA was incubated (15 min, 37 °C) with increasing concentrations of CR-VI+, in 20 mM Tris-HCl (pH 8.5), 1 mM DTT, 10% glycerol and 30 mM NaCl. The reaction mixtures were spotted onto nitrocellulose (GE Healthcare) using a manifold-1 dot-blot apparatus (Whatman) and washed with 20 mM Tris-HCl (pH 8.5), 1 mM DTT and 50 mM NaCl. Membrane-bound RNA was quantified by phosphor-imaging. Dissociation constants (*K*_D_s) were determined using Hill slope curve fitting (Prism).

### NTPase assay

Samples combining CR-VI+ (4 μM) with 0.5 μCi [α-^32^P]-GTP or 0.5 μCi [α-^32^P]-ATP in 40 mM Tris-HCl (pH 8.5), 1 mM DTT and 5 mM MgCl_2_ were incubated at 37 °C. Reactions were stopped by adding an equal volume of formamide/EDTA gel-loading buffer, and hydrolysis products were separated over a 20% polyacrylamide/8 M urea gel before phosphor-imaging.

## Additional information

**Accession codes:** Coordinates and structure factors are deposited in the Protein Data Bank under accession codes 4UCI, 4UCL, 4UCJ, 4UCK, 4UCZ, 4UCY and 4UD0.

**How to cite this article:** Paesen, G. C. *et al*. X-ray structure and activities of an essential *Mononegavirales L*-protein domain. *Nat. Commun.* 6:8749 doi: 10.1038/ncomms9749 (2015).

## Supplementary information

Supplementary Information Supplementary Figures 1-8 (PDF 1224 kb)

## Data Availability

Protein Data Bank
4UCI
4UCJ
4UCK
4UCL
4UCY
4UCZ
4UD0
