# Supplementary material for: X-ray structure and activities of an essential Mononegavirales L-protein domain
Source: Nat Commun. 2015 Nov 9;6:8749. doi: 10.1038/ncomms9749 (PMC4659945; doi:10.1038/ncomms9749)
Supplement: Supplementary Information — Supplementary Figures 1-8 (PDF 1224 kb) [file 41467_2015_BFncomms9749_MOESM819_ESM.pdf]

SUPPLEMENTARY INFORMATION

Supplementary Figure 1 : Recombinant CR-VI+ and dimer structure

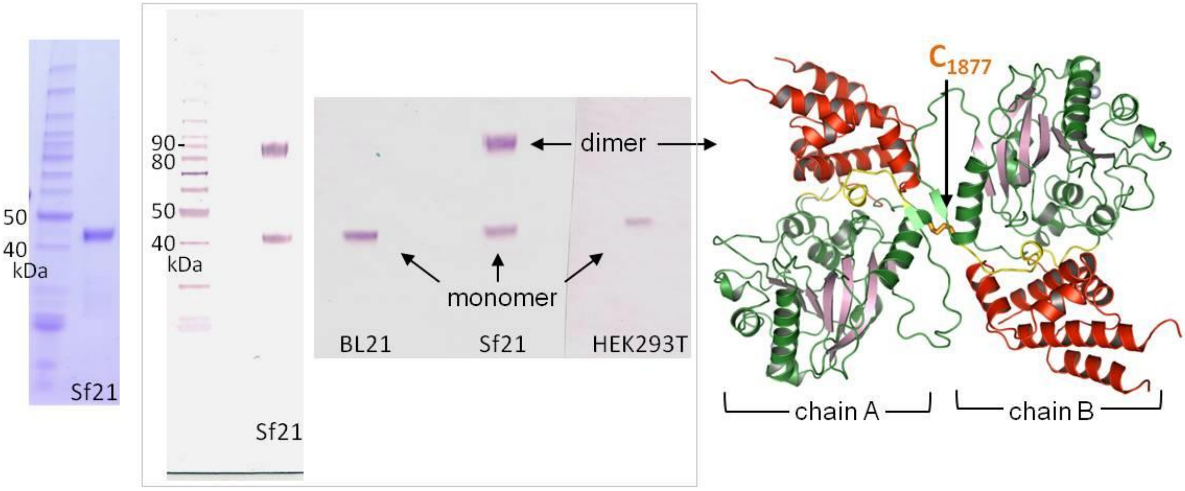

Left: Coomassie-stained, reducing SDS-gel showing insect-cell derived CR-VI+ protein (2nd lane) running in between the 40 and 50 kDa protein marker (BenchMark, Invitrogen; 1st lane). The protein was purified using a single (chelating) chromatography step. Middle: Western blots of non-reducing SDS-gels. In insect cells (Sf21), CR-VI+ is produced as a mixture of monomers and dimers, whilst bacteria (BL21) and mammalian cells (HEK293T) only express monomers, suggesting that the dimeric form is an expression artefact. The blot at the left shows the non-reduced insect-cell derived protein next to the BenchMark protein ladder, with the dimeric form running between the 80 and 90 kDA marker. Yields from insect cells were much higher than those from bacteria and mammalian cells, and most experiments were therefore carried out with *Sf21*-derived CR-VI+*.* As the protein tended to aggregate at each purification step, no attempts were made to separate monomers from dimers, but reducing agent was added to reaction buffers used in activity studies. Right: Crystal structure of dimeric CR-VI+ obtained from *Sf*21 cells, with the *+* domain in red, and the CR-VI domain in pink (β-strands) and green (helices and loops), except for $\lambda_{1650\text{-}1666}$ (yellow). The arrow indicates the inter-chain disulphide-bond linking the C_{1877} residues (orange).

Supplementary Figure 2 : Alignment of the C-termini of *L* proteins

PARAMYXOVIRIDAE

human Metapneumovirus	1894	-NLGNAEIKKLIKVTGYMLVS-	2003
avian Metapneumovirus	1984	-NLNSSELKKLVKVTGYILST-	2003
murine Pneumonia virus	2018	-SVSTSELKKVIKVTGILFRS-	2037
human Respiratory Syncytial virus	2142	-SLTTNELKKLIKITGSLLYN-	2161
bovine Respiratory Syncytial virus	2138	-SLTTNELKKLIKVTGSVLYS-	2157
Sendai virus	2184	-RFLTKEIKILMKILGAVKMF-	2203
Tupaia virus	2250	-AIITKEIKLWWKLLGYSYLL-	2269
Measles virus	2262	-KVTVKETKEWYKLVGYSALI-	2281
Rinderpest virus	2162	-KLTTKEIKEWFKLIGYSALI-	2181
Canine Distemper virus	2162	-QLETKEIKEWFKLLGYSALI-	2181
Narivavirus	2182	-HMTPREIKLWWKAISYSFLV-	2201
Mossman virus	2182	-PITTPEAKMWWKAIGYSVLM-	2201
Nipah virus	2225	-DLSNREVKIWWKIIGYISII-	2244
J virus	2182	-NLSTPEVKIWWKIVGYSVLY-	2201
Beilong virus	2150	-PLPTAEVKIWWKIVGYSVLH-	2169
Tailam virus	2150	-PLQTAEVKVWWKIVGYSVLH-	2169
Newcastle Disease virus	2178	-YLTRAQQKFYMKTIGNAAKG-	2197
Tuhoko virus 1	2224	-YLSRAQQKRVWKSVGSVILT-	2243
Tuhoko virus 2	2218	-EFQRSEQKKLWKNIGCIAFI-	2237
Tuhoko virus 3	2220	-LLSRAEQKTVWKQIGAINLV-	2239
Mapuera virus	2206	-QFSRSQQKQIWKAIGCSALV-	2225
human Parainfluenza virus 3	2188	-WFLTKEVKILMKLIGGAKLL-	2207
human Parainfluenza virus 4b	2222	-KLSRPKQKQIWKILGCTLFV-	2241
porcine Rubulavirus	2206	-FLSRPMQKRVWKTIGCALME-	2225
simian virus 41	2218	-PIHRSYQKRIWKALGSVIYC-	2237
Mumps virus	2212	-LLNRAYQKRIWKAIGCVIYC-	2231
Menangle virus	2220	-FLDRPTQKRIWKSVGSVILE-	2239
Tioman virus	2222	-KLDRSEQKRIWKAIGSVILS-	2241
Fer-de-lance virus	2159	-EITNRDKKKLFKLIGSAFYF-	2178
avian Paramyxovirus 4	2185	-NKARDFFKRRLKLVGFSLCG-	2204
avian Paramyxovirus 5	2241	-TLTRHEVKLFIKYLGSILKG-	2260
avian Paramyxovirus 7	2206	-MLSRSETKLLIKVLSAAWKG-	2225
avian Paramyxovirus 8	2218	-VLTRAEVKVCIKFLGAIIKL-	2237

FILOVIRIDAE

Sudan Ebolavirus	2182	-RMSDAEIKLMDRLTSLVNMF-	2201
Zaire Ebolavirus	2185	-RMQDSEVKLIERLTGLLSLF-	2204
Tai Forest Ebolavirus	2185	-RMQDSEIKLIDRLTGLLSLC-	2204
Reston Ebolavirus	2183	-RLRDAEIKLIERLTGLMRFY-	2202
Marburg virus	2300	-NTKIAEQKLLNRVIGYILFF-	2319
Lloviu Cuevavirus	2173	-RTDQAERKLLNRLVGLVQFF-	2192

Alignment of members of the *Paramyxo-* and *Filoviridae*, showing the conserved K-K-G motif (the second lysine in the motif is replaced by an arginine in *Filoviridae*). Members of the *Pneumovirinae* subfamily are listed in blue. The motif is not obvious in *Rhabdoviridae, Nyaviridae* or *Bornaviridae L* proteins.

Supplementary Figure 3 : Plasticity of the *+* domain at the height of the $\alpha^{+}2$-$\alpha^{+}3$ transition

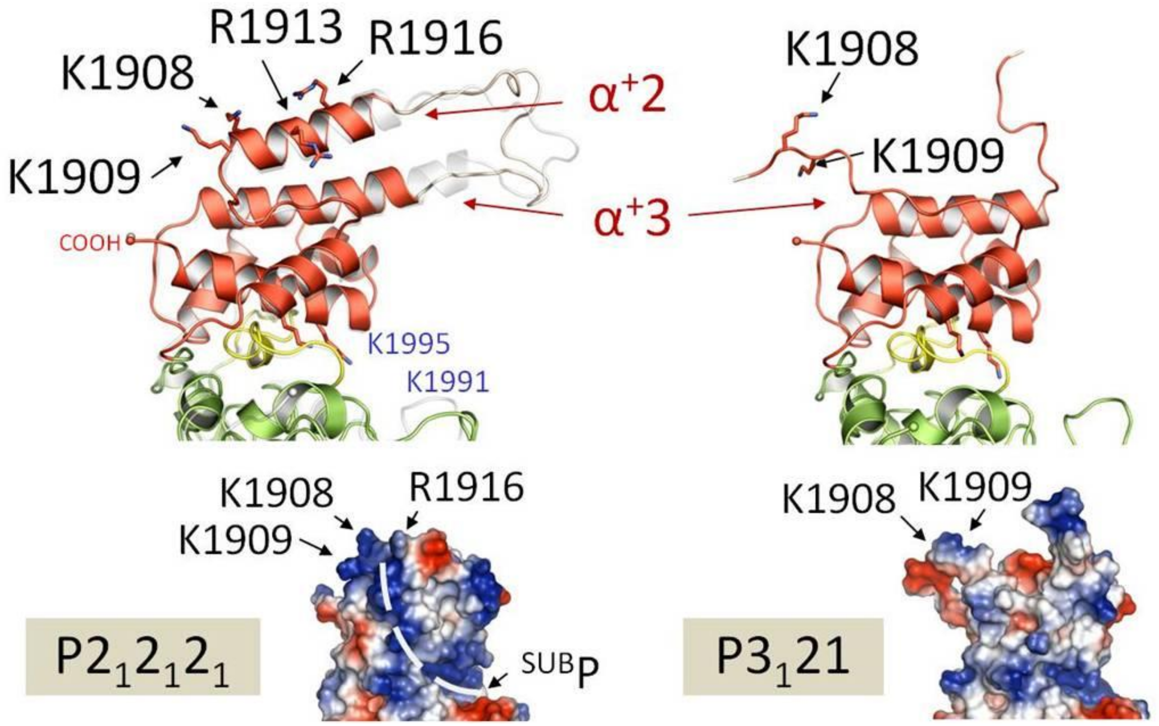

The cartoons show the varying lengths of the helices in $P2_12_12_1$ structures (left; red helices correspond to PDB 4UCI, white helices to 4UCY) and the complete absence of $\alpha^{+}2$ in a $P3_121$ structure (right; 4UD0). The basic residues of $\alpha^{+}2$ are shown as sticks, as are K_{1991} and K_{1995} of the K-K-G motif. The loops linking the helices in the $P2_12_12_1$ structures are representative since the electron-density is somewhat diffuse. The cartoons further illustrate the close connection of $\lambda_{1650\text{-}1666}$ (in yellow) to the *+* domain, and its disengagement from CR-VI (green). The dramatic effect of the plasticity on the surface characteristics of the *+* domain is shown underneath the cartoons. The surfaces are coloured according to their basic (blue) or acidic (red) charges. The dashed white line follows a basic region extending from the RNA-binding site (^{SUB}P) up to the $\alpha^{+}2$ helix in PDB 4UCI, which dissolves as the helix unwinds.

Supplementary Figure 4 : Structural alignment to flavivirus MTases

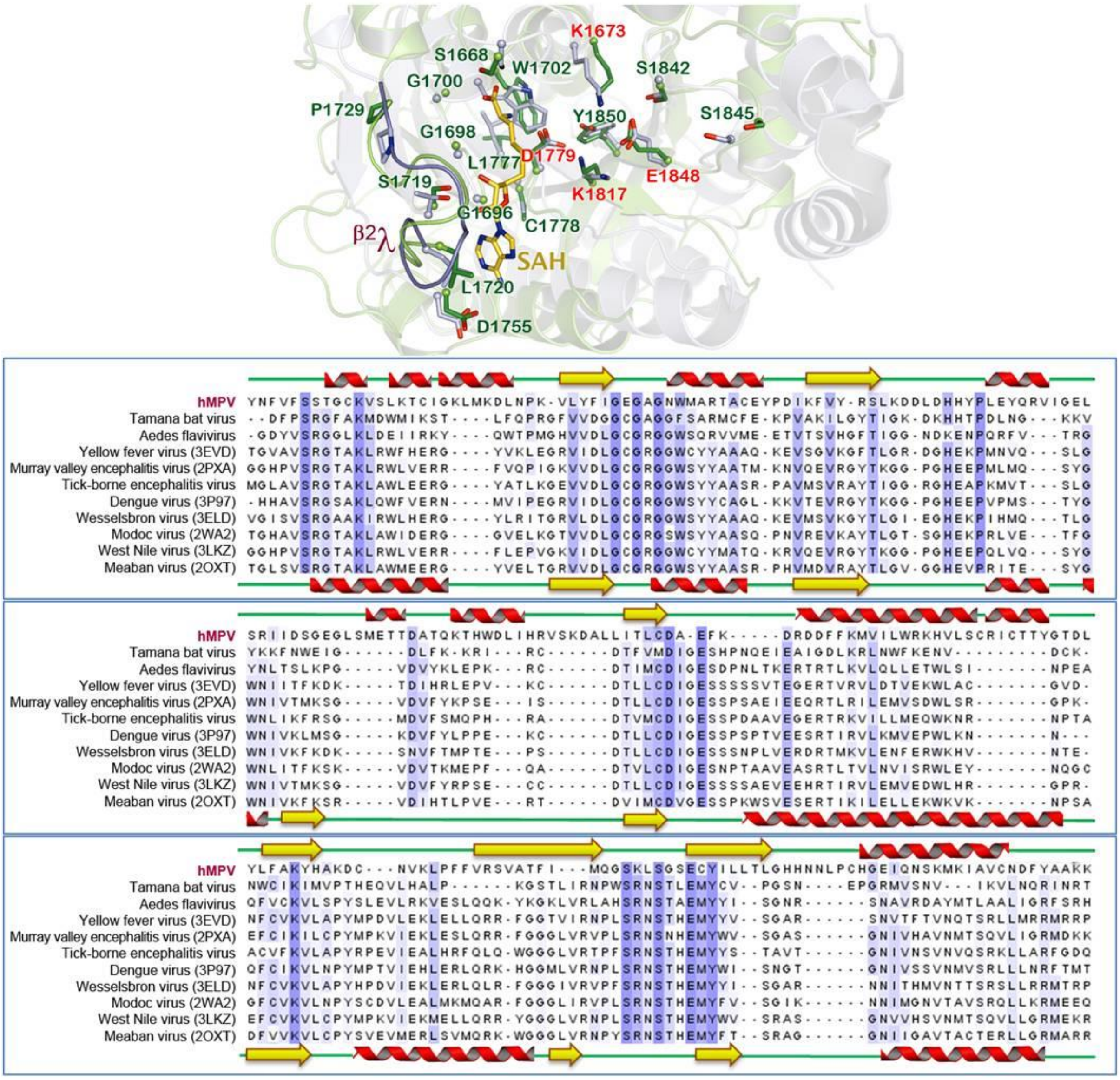

Structural overlay of CR-VI+ (green) onto the Yellow Fever virus MTase (PDB 3EVF; light blue), with identical residues (which predominantly cluster around ^{SAM}P) shown as sticks. Centrally to the K-D-K-E tetrad (red residue labels) lays tyrosine (Y_{1850}), a conserved residue of Rrmj-type MTases. The long $^{\beta 2}\lambda$ loop is highlighted. The alignment corresponding to the overlay is shown underneath, and includes additional flavivirus MTases. PDB codes are given between brackets, where available. The alignment covers residues 1663-2005 of hMPV *L* and 51-247 of the flavivirus sequences (PDB-files numbering). The secondary structure elements of CR-VI+ are given on top of the alignment, those of flavivirus MTases at the bottom (loops are in green, β-strands in yellow and α-helices in red).

Supplementary Figure 5 : The role of ^{NS}P

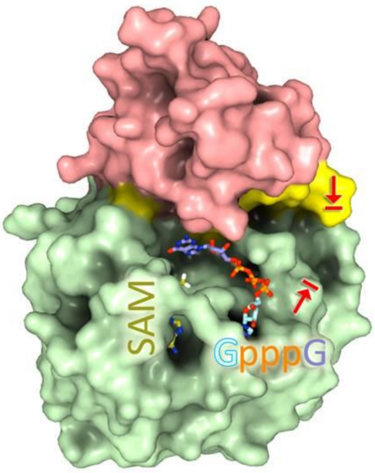

Surface presentation of CR-VI+ (colour scheme as in Figure 6a). Although co-crystals of CR-VI+ with cap analogues could not be obtained, **G**pppG can be modelled into the protein with **G** (light blue) occupying ^{NS}P and the N1 nucleotide (purple) occupying ^{SUB}P. In other MTases, a narrow, high-affinity cap-binding site is found in the space between the arrows.

Supplementary Figure 6 : Involvement of the long, N-terminal loop in GTPase activity

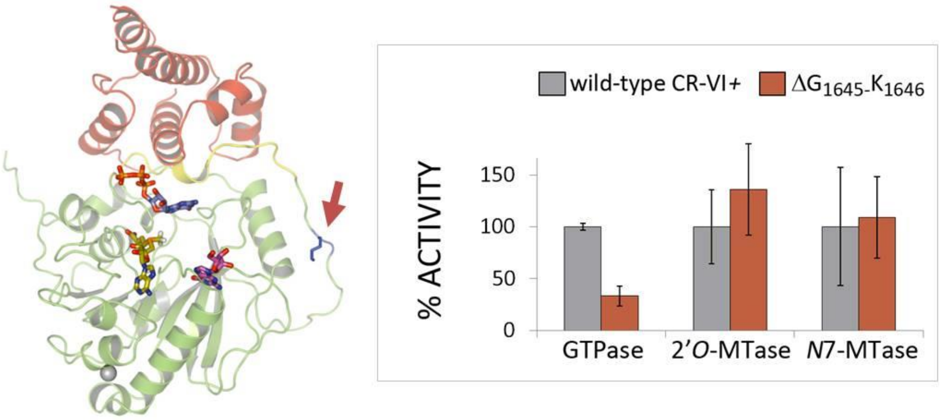

Removing N-terminal-loop residues G_{1645} and K_{1646} (indicated by the arrow in the structure) resulted in a marked reduction in GTPase activity, without affecting the MTase reactions, suggesting the mutation did not alter the overall structure (the activities of wild-type CR-VI+ were set at 100%; measurements were carried out as in Figs. 3b and 4; the bars and error bars correspond to the mean values from 3 measurements and their standard deviations, respectively). The N-terminal loop may be directly involved in the GTPase reaction (*e.g.* by folding over a nearby site containing the substrate), or indirectly (*e.g.* by restricting the flexibility of $\lambda_{1650-1666}$ and thus the movement of the + domain relative to the MTase domain.

Supplementary Figure 7 : Synthetic gene and primers used for mutagenesis

```
atggctctgctgacccctatcccctcccctatggtcaacctgacccaagtgatcgacccc
 M   A   L   L   T   P   I   P   S   P   M   V   N   L   T   Q   V   I   D   P      1618
accgagcagctggcttacttccccaagatcaccttcgagcgcctgaagaactacgacact
 T   E   Q   L   A   Y   F   P   K   I   T   F   E   R   L   K   N   Y   D   T      1638
tcctccaactacgctaagggcaagctgacccgtaactacatgatcctgctgccctggcag
 S   S   N   Y   A   K   G   K   L   T   R   N   Y   M   I   L   L   P   W   Q      1658
cacgtgaaccgttacaacttcgtgttctccagcaccggttgcaaggtgtcactcaagacc
 H   V   N   R   Y   N   F   V   F   S   S   T   G   C   K   V   S   L   K   T      1678
tgcatcggcaagctgatgaaggacctgaaccccaaggtgctgtacttcatcggcgagggt
 C   I   G   K   L   M   K   D   L   N   P   K   V   L   Y   F   I   G   E   G      1698
gctggcaactggatggctcgtaccgcttgcgagtaccccgacatcaagttcgtgtaccgt
 A   G   N   W   M   A   R   T   A   C   E   Y   P   D   I   K   F   V   Y   R      1718
tccctgaaggacgacctcgaccaccactacccctcgagtaccagcgtgtgatcggcgag
 S   L   K   D   D   L   D   H   H   Y   P   L   E   Y   Q   R   V   I   G   E      1738
ctgtcccgtatcatcgactccggcgagggcctgtctatggaaaccaccgacgctacccaa
 L   S   R   I   I   D   S   G   E   G   L   S   M   E   T   T   D   A   T   Q      1758
aagacccactgggacctgatccaccgtgtgtccaaggacgccctgctgatcaccctgtgc
 K   T   H   W   D   L   I   H   R   V   S   K   D   A   L   L   I   T   L   C      1778
gacgctgagttcaaggaccgtgacgacttcttcaagatggtcatcctgtggcgcaagcac
 D   A   E   F   K   D   R   D   D   F   F   K   M   V   I   L   W   R   K   H      1798
gtgctgtcctgccgtatctgcaccacctacggcaccgacctgtacctgttcgctaagtac
 V   L   S   C   R   I   C   T   T   Y   G   T   D   L   Y   L   F   A   K   Y      1818
cacgctaaggactgcaacgtgaagctgcccttcttcgtgcgttccgtggctaccttcatc
 H   A   K   D   C   N   V   K   L   P   F   F   V   R   S   V   A   T   F   I      1838
atgcaaggttccaagctgtccggttccgagtgctacatcctgctcaccctgggtcaccac
 M   Q   G   S   K   L   S   G   S   E   C   Y   I   L   L   T   L   G   H   H      1858
aacaacctgccctgccacggcgagatccagaacagcaagatgaagatcgccgtgtgcaac
 N   N   L   P   C   H   G   E   I   Q   N   S   K   M   K   I   A   V   C   N      1878
gacttctacgctgctaagaagctggacaacaagagcatcgaggctaactgcaagtccctg
 D   F   Y   A   A   K   K   L   D   N   K   S   I   E   A   N   C   K   S   L      1898
ctgtccggcctgcgtatccccatcaacaagaaggaactcaaccgtcagcgtcgcctgctg
 L   S   G   L   R   I   P   I   N   K   K   E   L   N   R   Q   R   R   L   L      1918
accctccagtccaaccactcctctgtggctaccgtgggcggttctaaggtcatcgagtct
 T   L   Q   S   N   H   S   S   V   A   T   V   G   G   S   K   V   I   E   S      1938
aagtggctcaccaacaaggccaacaccatcatcgactggctcgagcacatcctgaactcc
 K   W   L   T   N   K   A   N   T   I   I   D   W   L   E   H   I   L   N   S      1958
cccaagggcgagctgaactacgacttcttcgaggctctcgagaacacctaccccaacatg
 P   K   G   E   L   N   Y   D   F   F   E   A   L   E   N   T   Y   P   N   M      1978
atcaagctcatcgacaacctgggcaacgctgaaatcaagaagttgatcaaggtcaccggc
 I   K   L   I   D   N   L   G   N   A   E   I   K   K   L   I   K   V   T   G      1998
tacatgctggtgtccaagaagtccggacaccatcaccatcatcactgatag
 Y   M   L   V   S   K   K   S   G   H   H   H   H   H   H                          2013
```

mutant	forward primer (from 5’ to 3’)	reverse primer (from 5’ to 3’)
Q1658A	TATAATGCAGTGGCTCACGTGAACCGTTACAACTTC	TATAATGCAGTGGCCCAGGGCAGCAGGATC
H1659A	ATATGCTCTTCAGCTGTGAACCGTTACAACTTCGTG	ATATGCTCTTCAAGCCTGCCAGGGCAGCAG
R1662A	ATATGCTCTTCAGCTTACAACTTCGTGTTCTCCAGCAC	ATATGCTCTTCAAGCGTTCACGTGCTGCCAGG
R1662E	ATATGCTCTTCAGAGTACAACTTCGTGTTCTCCAGCAC	ATATGCTCTTCACTCGTTCACGTGCTGCCAGG
R1662Q	ATATGCTCTTCACAGTACAACTTCGTGTTCTCCAGCAC	ATATGCTCTTCACTGGTTCACGTGCTGCCAG
F1665A	TATAATGCAGTGGCTTCCAGCACCGGTTGC	TATAATGCAGTGGCCACGAAGTTGTAACGGTTCAC
S1668A	TATAATGCAGTGGCTAGCACCGGTTGCAAGG	TATAATGCAGTGGCGAACACGAAGTTGTAACGGTTC
S1669A	TATAATGCAGTGGCTACCGGTTGCAAGGTGTC	TATAATGCAGTGGCGGAGAACACGAAGTTGTAACG
T1670A	ATATGCTCTTCAGCTGGTTGCAAGGTGTCACTCAAG	ATATGCTCTTCAAGCGCTGGAGAACACGAAGTTGTAAC
K1673A	TATAATGCAGTGGCTGTGTCACTCAAGACCTGCATC	TATAATGCAGTGGCGCAACCGGTGCTGG
E1697C	TATAATGCAGTGGCGGTGCTGGCAACTGGATG	TATAATGCAGTGGCAGCCGATGAAGTACAGC
H1727A	TATAATGCAGTGGCTTACCCCCTCGAGTACCAG	TATAATGCAGTGGCGTGGTCGAGGTCGTCC
D1779A	ATATGCTCTTCAGCTGCTGAGTTCAAGGACCGTG	ATATGCTCTTCAAGCGCACAGGGTGATCAGC
D1779Q	ATATGCTCTTCACAGGCTGAGTTCAAGGACCGTG	ATATGCTCTTCACTGGCACAGGGTGATCAGCAG
E1781A	TATAATGCAGTGGCTGCTTTCAAGGACCGTGACGAC	TATAATGCAGTGGCGTCGCACAGGGTG
E1781Q	TATAATGCAGTGGCTCAGTTCAAGGACCGTGACGAC	TATAATGCAGTGGCGTCGCACAGGGTG
R1785A	TATAATGCAGTGGCTGACGACTTCTTCAAGATGGTCATC	TATAATGCAGTGGCGTCCTTGAACTCAGCGTCG
K1817A	TATAATGCAGTGGCTTACCACGCTAAGGACTGCAAC	TATAATGCAGTGGCAGCGAACAGGTACAGGTCG
K1817Q	TATAATGCAGTGGCTCAATACCACGCTAAGGACTGCAAC	TATAATGCAGTGGCGAACAGGTACAGGTCG
K1821Q	ATATGCTCTTCACAGGACTGCAACGTGAAGCTG	ATATGCTCTTCACTGAGCGTGGTACTTAGCGAACAGG
S1842A	ATATGCTCTTCAGCTAAGCTGTCCGGTTCCGAG	ATATGCTCTTCAAGCACCTTGCATGATGAAGGTAGCC
K1843A	ATATGCTCTTCAGCTCTGTCCGGTTCCGAGTGCTAC	ATATGCTCTTCAAGCGGAACCTTGCATGATGAAGG
E1848A	ATATGCTCTTCAGCTTGCTACATCCTGCTCACCC	ATATGCTCTTCAAGCGGAACCGGACAGCTTG
C1877A	TATAATGCAGTGGCTAACGACTTCTACGCTGCTAAGAAG	TATAATGCAGTGGCCACGGCGATCTTCATCTTG
K1991E	ATATGCTCTTCAGAGAAGTTGATCAAGGTCACCG	ATATGCTCTTCACTCGATTTCAGCGTTGCCC

K1991Q	ATATGCTCTTCACAGAAGTTGATCAAGGTCACCG	ATATGCTCTTCACTGGATTTCAGCGTTGCCC
K1992Q	ATATGCTCTTCACAGTTGATCAAGGTCACCGG	ATATGCTCTTCACTGCTTGATTTCAGCGTTGCC
K1995Q	ATATGCTCTTCACAGGTCACCGGCTACATG	ATATGCTCTTCACTGGATCAACTTCTTGATTTCAGCG
K1995E	ATATGCTCTTCAGAGGTCACCGGCTACATG	ATATGCTCTTCACTCGATCAACTTCTTGATTTCAGCG
K1991Q/K1992Q	TATAATGCAGTGGCAGTTGATCAAGGTCACCGG	TATAATGCAGTGGCTGGATTTCAGCGTTGCC
K1991Q/K1995Q	TATAATGCAGTGATCCAGAAGTTGATCCAGG	TATAATGCAGTGATTTCAGCGTTGCCCAG
K1991Q/K1992Q/K1995Q	TATAATGCAGTGGCAGTTGATCCAGGTCACCGG	TATAATGCAGTGGCTGGATTTCAGCGTTGCC
ΔG1645K1646	TATAATGCAGTGGCTAAGCTGACCCGTAACTACATGATC	TATAATGCAGTGGCGTAGTTGGAGGAAGTGTCG

The nucleotide sequence of the synthetic gene for the CR-VI+ domain is shown on top, with the corresponding protein sequence underneath. Residues that are not in the original hMPV *L* protein (the start methionine, the adjacent alanine, and the oligo-histidine-tag) are in bold and italic. The table lists the primers used to generate mutants. The *Bts*I and *BspQ*I recognition sequences are highlighted in gray and yellow, respectively.

Supplementary Figure 8 : Electron density map

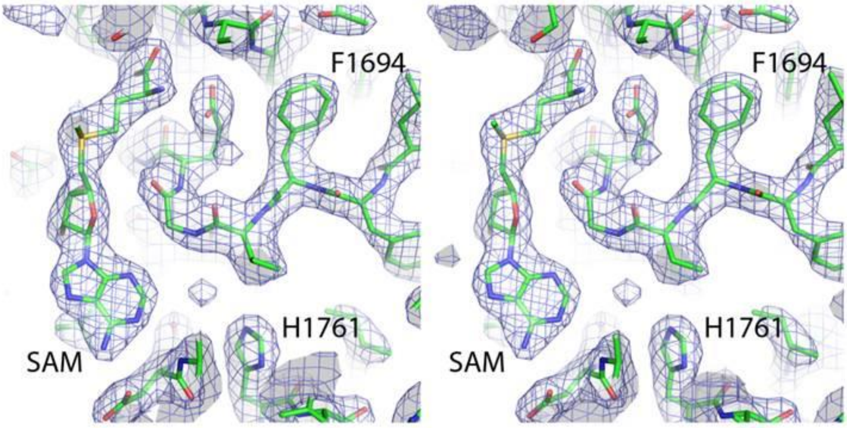

The stereo image shows the electron density of the SAM-binding site of the CR-VI+ structure (PDB: 4UCI, chain A). The 2Fo-Fc map was contoured at 1.5 sigma.
